# Environmental Fate and Effects of Road Run-Off

**DOI:** 10.1007/s00244-021-00906-3

**Published:** 2022-01-03

**Authors:** Patricia L. Gillis, Joanne L. Parrott, Paul Helm

**Affiliations:** 1 Aquatic Contaminants Research Division, Environment and Climate Change Canada, Burlington, ON Canada; 2 Ontario Ministry of the Environment, Conservation and Parks, Toronto, ON Canada

Anywhere that there are transportation corridors, there is the potential for runoff of materials that accumulate or are deposited on roads. Whether and how that runoff affects ecosystem health depends on a number of abiotic and biotic factors, including the chemical composition of the runoff and the sensitivity of the biota in the receiving environment. With growing populations worldwide leading to increased urbanization, urban pollution sources such as road runoff present a complex problem to scientists and resource managers. The complex mixture of pollutants associated with runoff includes particles released by vehicle wear from tires and pavement (Baensch-Baltruschat et al. 2020), polycyclic aromatic hydrocarbons and other hydrocarbons deposited from exhaust and fuel/oil spills (Markiewicz et al. 2017), a wide range of metals associated with automotive traffic (Huber et al. 2019), as well as chemical additives and modifiers (Hou et al. 2019). In addition, in temperate regions, road runoff in winter can contain the salts, brines and sand applied to road surfaces for road maintenance (Hintz and Relyea 2019).

During rain events or snow melt, any chemicals and particles associated with road runoff can be washed directly into ditches, wetlands and waterways or transported through storm water systems to larger waterbodies. An understanding of the transport and fate of road-associated contaminants including the re-distribution through air to terrestrial environments and wash-off into aquatic systems is required to characterize exposure and assess the potential risk to terrestrial and aquatic organisms (Chen et al. 2016). Contaminants originating from road runoff can be deposited to surfaces, may be bioaccumulated directly or partition to sediments, or dissolve in surface waters. The properties of chemicals and their reactivity in the aquatic environment will influence the exposure of organisms to road-associated contaminants, whether they cause direct toxic effects and whether accumulation in tissues and the food chain is possible. These pathways present many fascinating and complex questions for environmental chemists and ecotoxicologists assessing road runoff. In addition, scientifically, road runoff is a perfect candidate for exploration with non-targeted analysis, toxicity identification and evaluation, and effect-directed analysis.

## Special Issue Articles

Within this context, this special issue of AECT presents a collection of 11 articles describing recent studies on the fate and effects of road runoff. Broadly speaking, the articles span the disciplines of environmental chemistry, environmental fate modeling and ecotoxicology. The road runoff constituents investigated include particles and compounds from tire wear, dust suppressants, as well as various de-icing salts and metals. The freshwater biota employed in ecotoxicity studies included fish, amphibians and a range of invertebrates. In this issue, the research articles were contributed from colleagues based in Australia, Canada, China, Finland, France, India and the USA. The major findings from these articles are highlighted below under the headings of “Characterization and Fate,” “Ecotoxicity” and “Mitigation.”

### Characterization and Fate

Recently, there have been growing interest and concern regarding tire wear particles and associated chemicals and how they affect aquatic ecosystems. Johannessen et al. (2022a) demonstrated that hexamethoxymethyl-melamine (HMMM), a compound which is used in the manufacture of tires and is included in the US Environmental Protection Agencies “High Production Volume Challenge Program” is discharged during rain events into urban watersheds in the Greater Toronto Area in Canada. In addition, in another contribution, Johannessen et al. (2022b) reported that two other tire wear compounds, 6PPD-quinone and 1,3-diphenylguanidine, are also present at µg/L concentrations in these urban watersheds and these authors predicted that for each hydrological event, the cumulative loads of these compounds entering urban surface water are in kg amounts. This is alarming as Tian et al. (2021) recently reported that 6PPD-quinone is toxic to Coho salmon at concentrations < 1 µg/L. Regarding metals, in addition to the known risks related to metals associated with automotive traffic, new information is available on additional routes of transport and the presence of emerging metals of concern being associated with road runoff. Siddiqui and Pandey (2022) demonstrated that the levels of nutrients and metals in atmospheric deposition and runoff from three cities draining to India’s Ganga River were highly correlated and that most metals were present at concentrations higher than previously reported, particularly during first flush. Lerat-Hardy et al. (2022) conducted high-frequency sampling to determine the concentrations of metals, both trace (e.g., copper, zinc) and rare earth elements (e.g., lanthanum, cesium) in runoff from France’s longest urban highway. They demonstrated the importance of capturing first flush after a rainfall to accurately characterize metal loads associated with a runoff event, especially for the rarer elements. Zhang et al. (2022) characterized a wide range of semi-volatile organic compounds (SVOCs), including phthalates and pesticides in addition to mono- and polycyclic aromatic hydrocarbons, in runoff from impervious surfaces in a Beijing suburb and concluded that localized land use type was the driving factor in the composition and concentrations of SVOCs in runoff.

### Ecotoxicity

Many compounds, elements and ions reported in road runoff in this special issue were found at levels that can harm aquatic organisms. Izzo et al. (2022) used laboratory exposures with sodium chloride to investigate whether salt-laden road runoff had the potential to affect juvenile salamanders. While neither species of the salamanders studied experienced acute toxicity at levels typically found in (the studied) streams, exceedances of the Water Quality Criteria for chloride established by the US Environmental Protection Agency in some salamander habitats indicate the potential for sublethal and indirect effects on wild salamander populations. Three other studies demonstrated negative effects of salt laden winter runoff on invertebrates, both in the laboratory and in their natural habitats. Gillis et al. (2022) reported that the acute toxicity of road runoff collected from a bridge during the winter to mussel larvae in the laboratory was consistent with reduced wild mussel abundance in the river under that highway bridge. Shenton et al. (2022) reported differences between the stream macroinvertebrate community composition in Australian sub-alpine habitats that receive salt inputs from road runoff and the areas that do not receive salt. They concluded that salt was responsible for the observed alterations in invertebrate communities as taxa such as Ephemeroptera that were less abundant where de-icing salts were used were the same taxa that exhibited salt sensitivity in laboratory tests. Moulding et al. (2022) evaluated the toxicities of various products used for winter road maintenance and whether the toxicity of these products to sensitive freshwater biota was affected by temperature. Significant differences in toxicity between de-icing products were reported, as well as increased toxicity to mayfly larvae at higher temperatures (15 °C) compared to lower temperatures (4–7 °C). Regarding toxicity of tire wear compounds and particles, Johannessen et al. (2022b) reported that the maximum levels of 6PPD-quinone in an urban river during storm events exceeded the previously reported LC50 for this compound for Coho salmon (Tian et al. 2021). However, Navarro et al. (2022) found that microplastics derived from tire rubber were not harmful to sediment-dwelling oligochaetes and insect larvae. Dust suppressants are also a concern as more than 65% of the world’s roads are unpaved. In their paper, Kunz et al. (2022) reported a wide range of toxicities of road dust suppressing compounds, observing more than a thousand-fold difference in fish acute LC50s.

### Mitigation

With growing urbanization, it is important to learn which mitigation measures will be most effective at reducing the transport of harmful constituents in road runoff, as this, along with managing the route of runoff to sensitive ecosystems, will reduce its negative impacts on the environment. Izzo et al. (2022) illustrated the seasonal differences in chloride levels in streams managed with storm water retention ponds compared to those without, and Zhang et al. (2022) recommended that SVOC-impacted runoff from some land uses such as gas stations and roads be collected in pipes for treatment rather than being allowed to travel overland to receiving waters. Finally, Gillis et al. (2022) reported that runoff from a bridge directed through a tile drain contained lower levels of chloride and was less toxic to mussels than runoff that flowed directly from a bridge deck drain into the receiving environment.

## Looking Ahead

Due to its complexity and the localized and seasonal differences in the composition of road runoff, as well as changing road maintenance practices that include new alternatives to road salt, much remains to be learned about the environmental fate and effects of road runoff. In addition, climate change is expected to increase the duration of dry events, followed by intense storm events, making road runoff pulses more episodic and potentially more harmful to aquatic organisms. Therefore, it is clear there are many more questions to ask and systems to study before we fully understand the risks that road runoff poses not only to ecosystem health but inevitably to human health as well.
